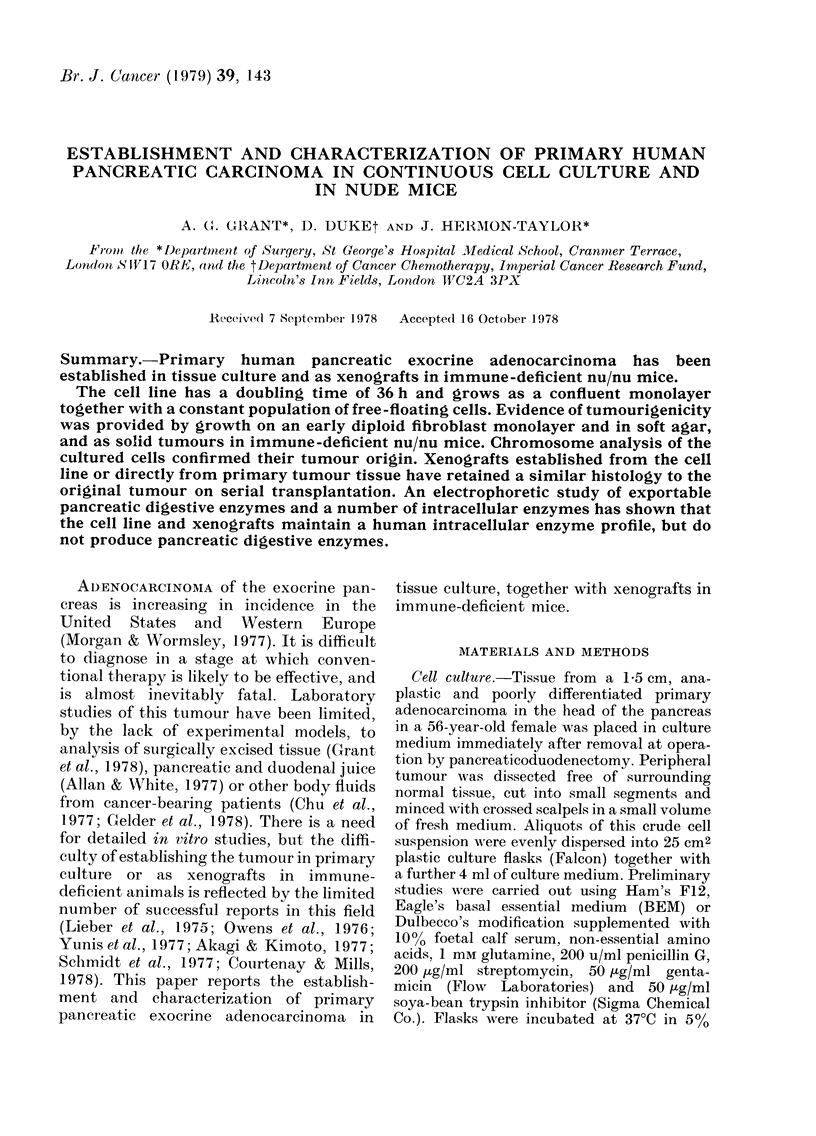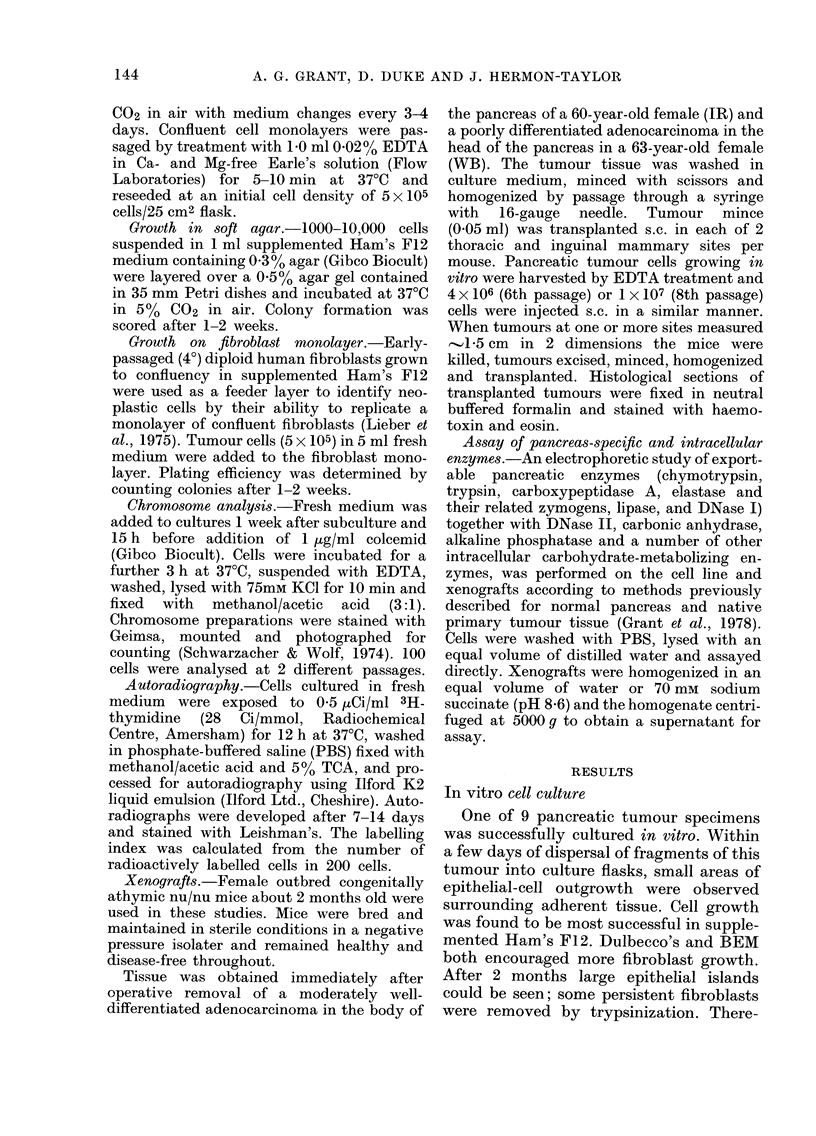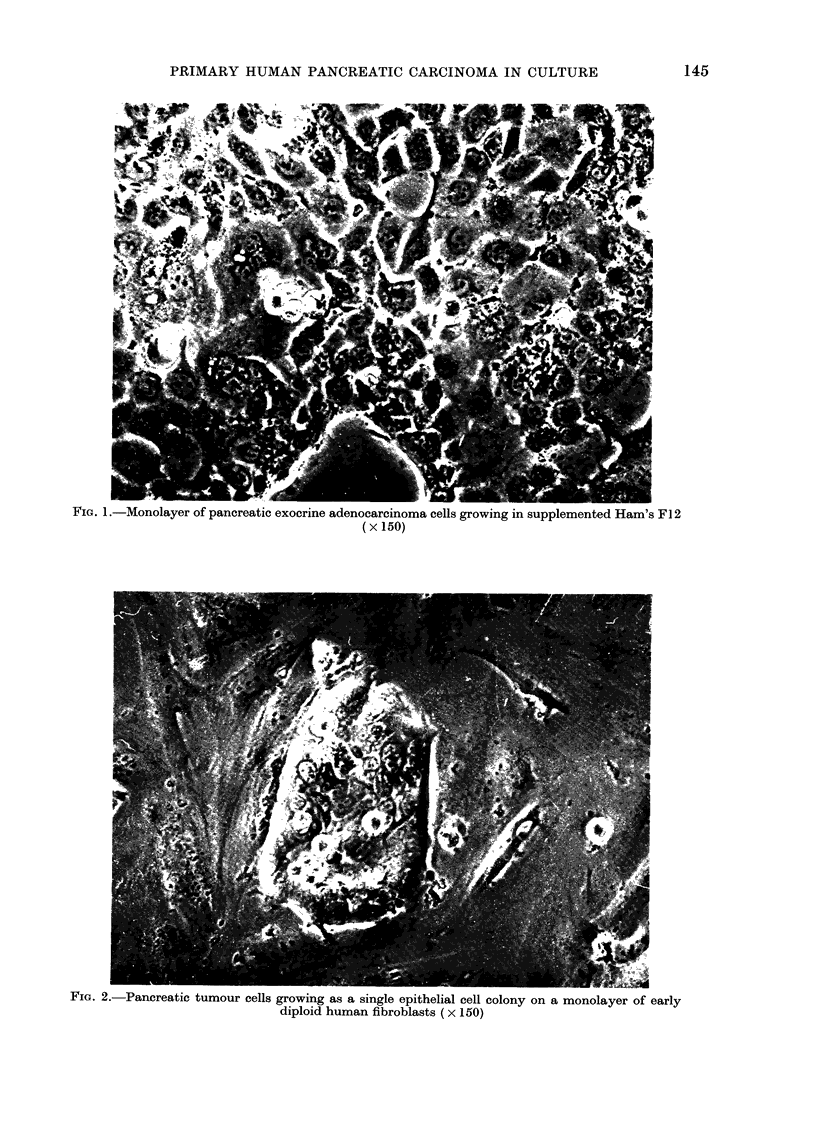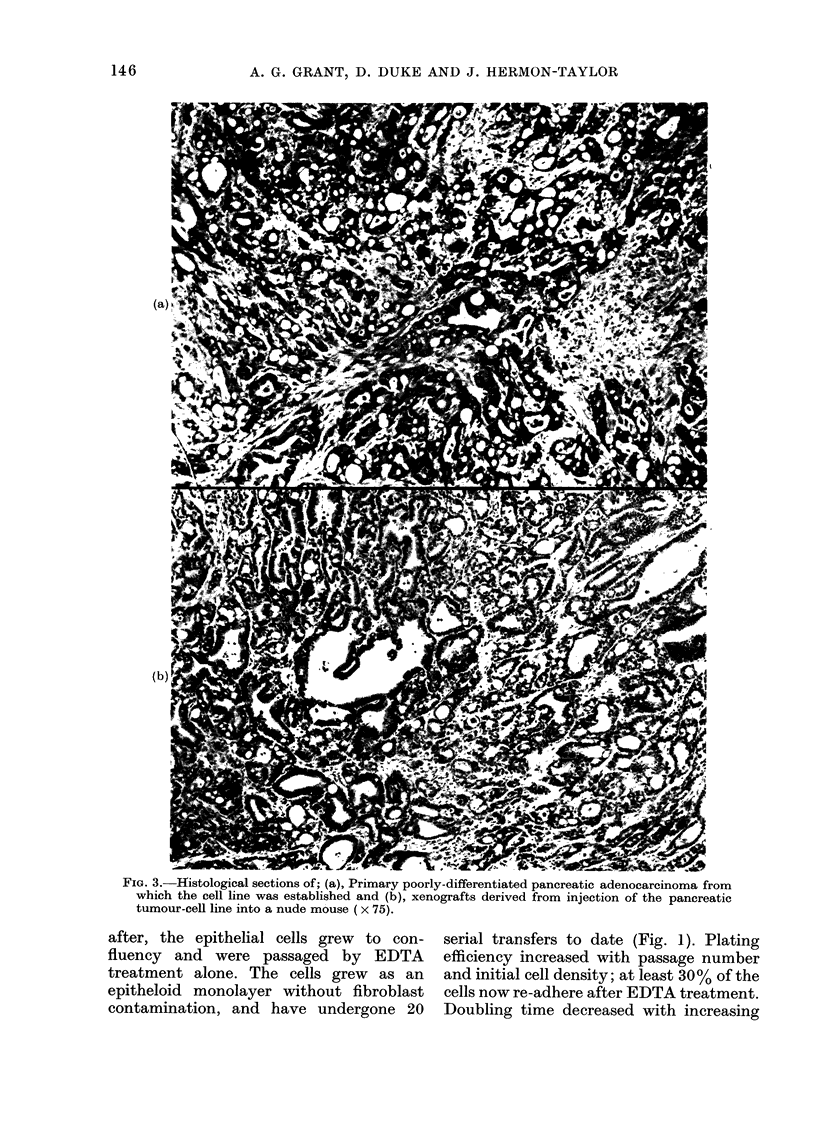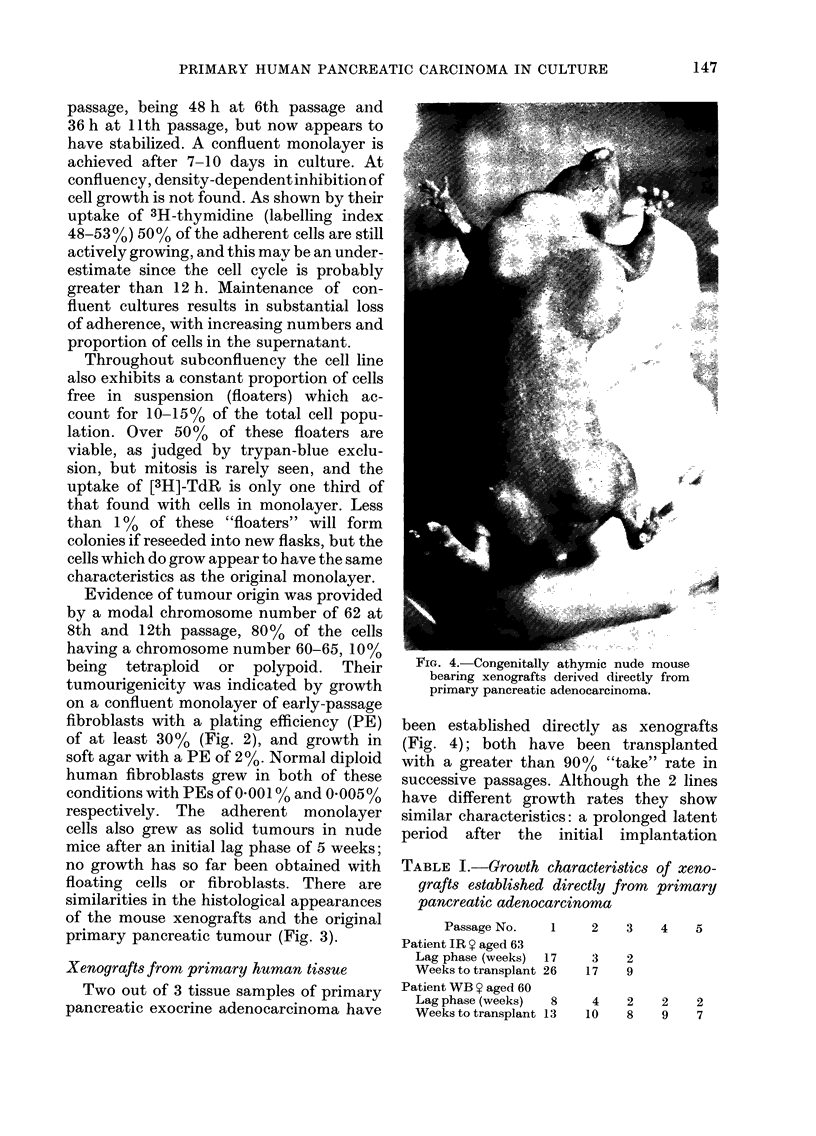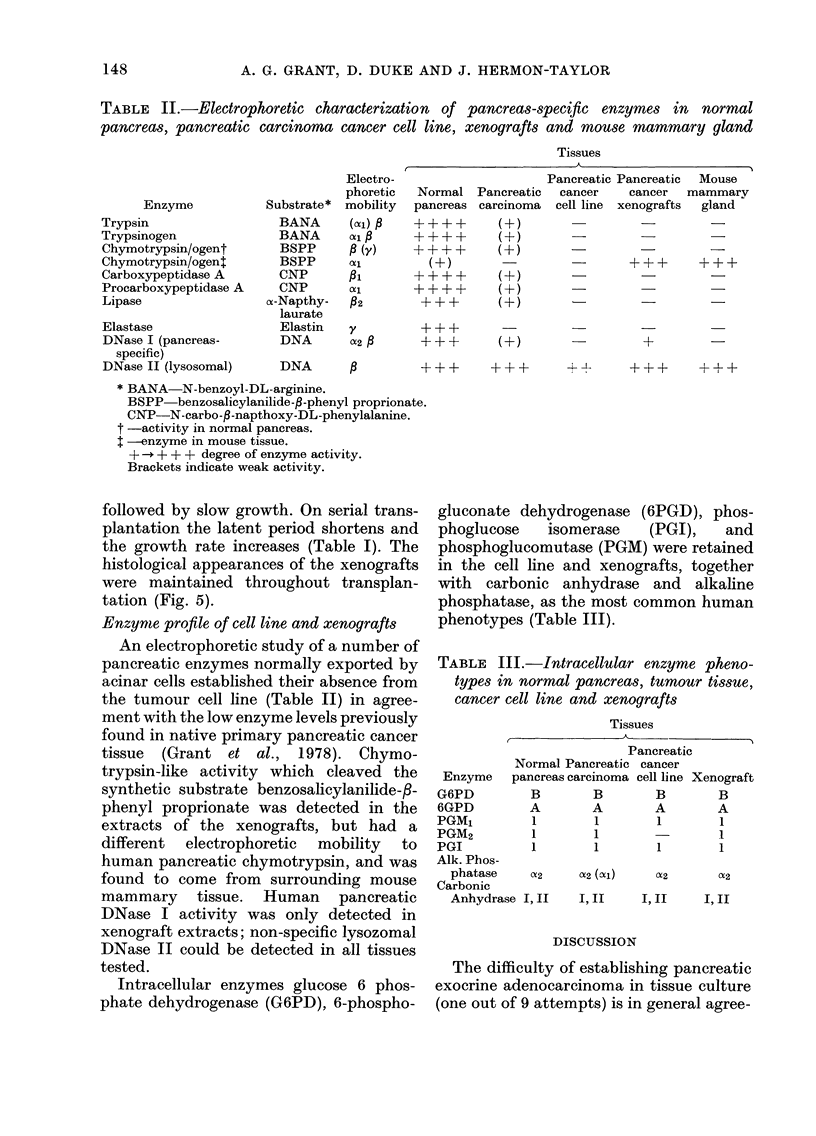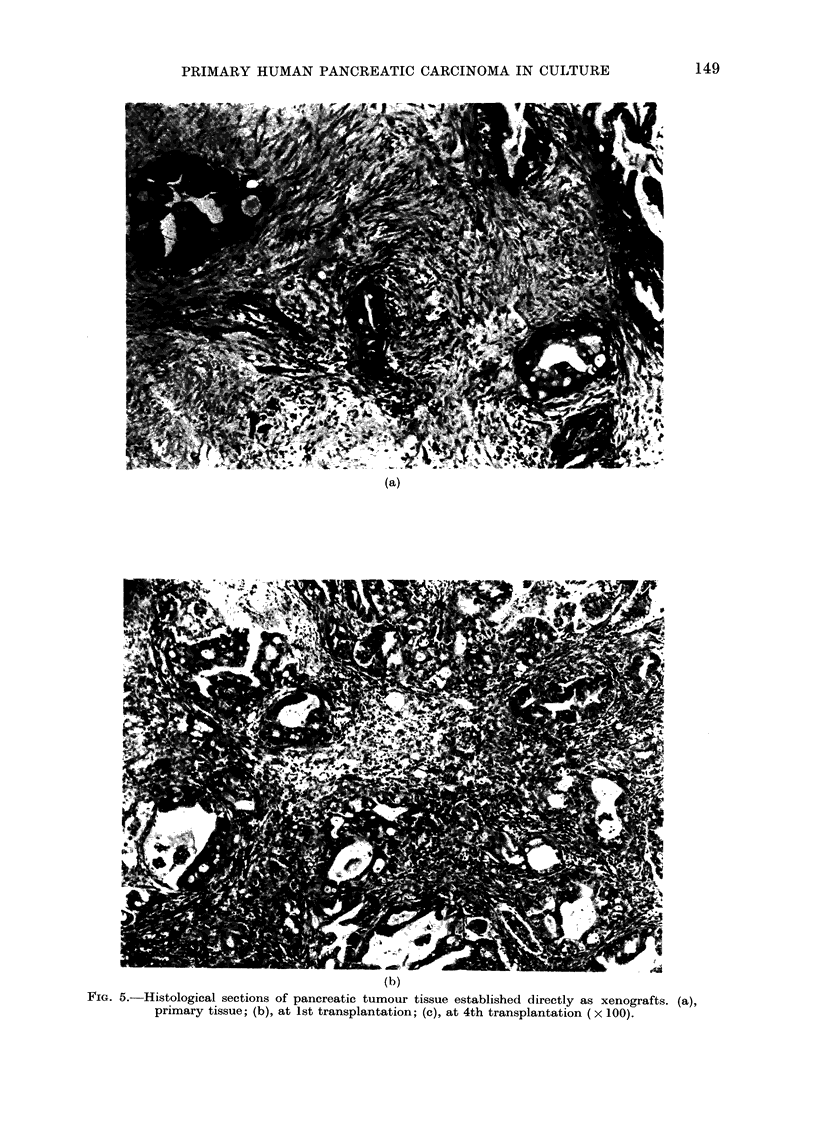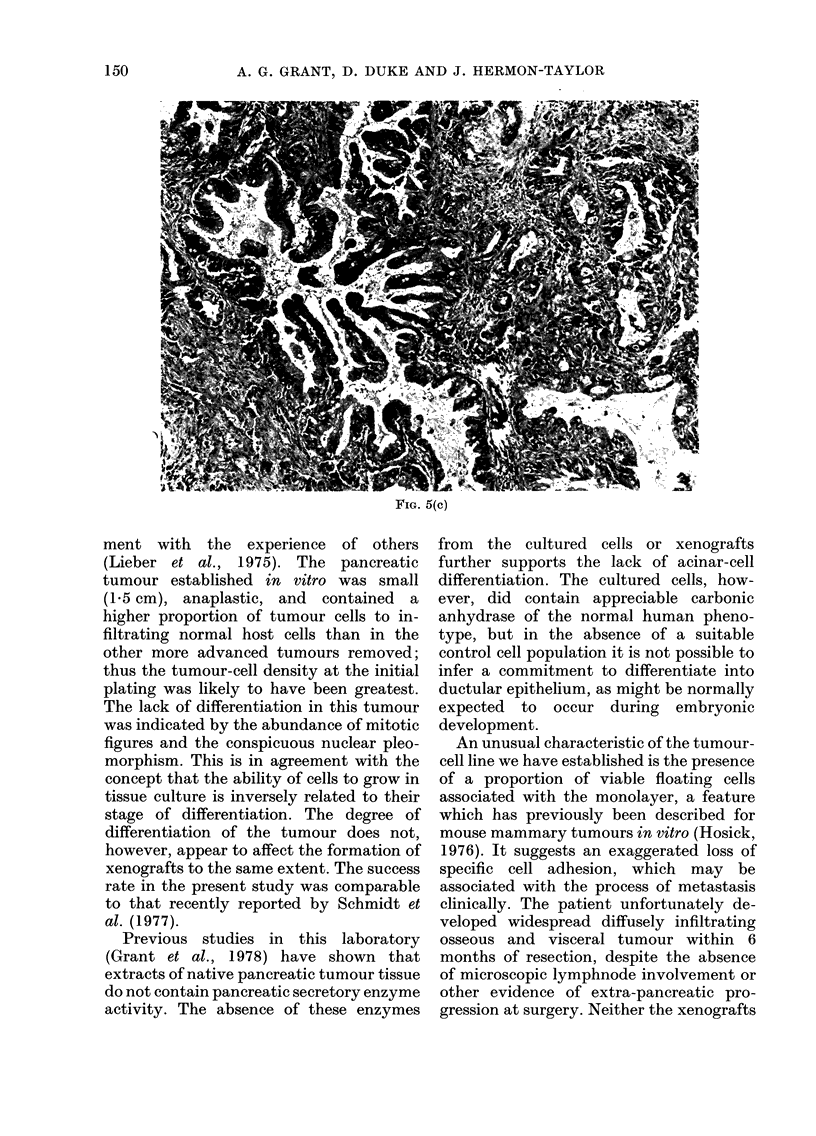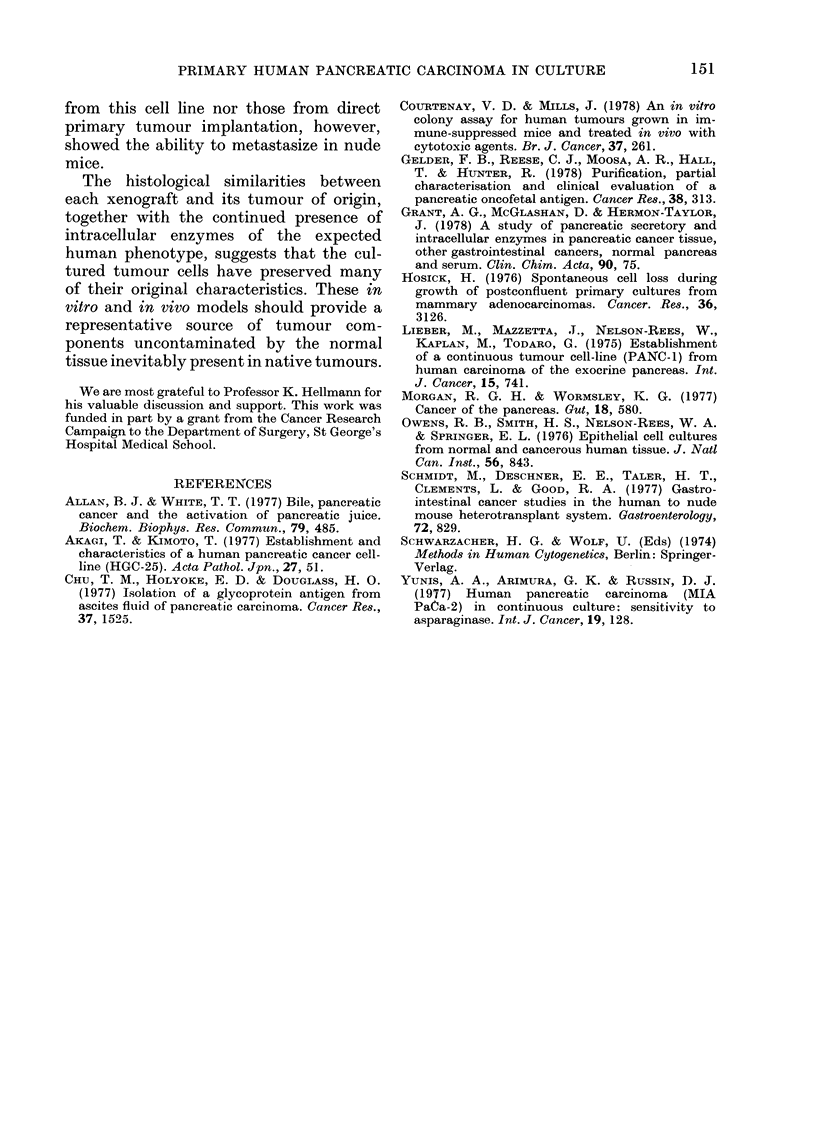# Establishment and characterization of primary human pancreatic carcinoma in continuous cell culture and in nude mice.

**DOI:** 10.1038/bjc.1979.24

**Published:** 1979-02

**Authors:** A. G. Grant, D. Duke, J. Hermon-Taylor

## Abstract

**Images:**


*Br. J. Cancer* (1979) **39**, 143

### ESTABLISHMENT AND CHARACTERIZATION OF PRIMARY HUMAN PANCREATIC CARCINOMA IN CONTINUOUS CELL CULTURE AND IN NUDE MICE

A. G. GRANT*, D. DUKE† AND J. HERMON-TAYLOR*

*From the *Department of Surgery, St George's Hospital Medical School, Cranmer Terrace, London SW17 0RE, and the †Department of Cancer Chemotherapy, Imperial Cancer Research Fund, Lincoln's Inn Fields, London WC2A 3PX*

Received 7 September 1978 Accepted 16 October 1978

**Summary.—Primary human pancreatic exocrine adenocarcinoma has been established in tissue culture and as xenografts in immune-deficient nu/nu mice.**

**The cell line has a doubling time of 36 h and grows as a confluent monolayer together with a constant population of free-floating cells. Evidence of tumourigenicity was provided by growth on an early diploid fibroblast monolayer and in soft agar, and as solid tumours in immune-deficient nu/nu mice. Chromosome analysis of the cultured cells confirmed their tumour origin. Xenografts established from the cell line or directly from primary tumour tissue have retained a similar histology to the original tumour on serial transplantation. An electrophoretic study of exportable pancreatic digestive enzymes and a number of intracellular enzymes has shown that the cell line and xenografts maintain a human intracellular enzyme profile, but do not produce pancreatic digestive enzymes.**

ADENOCARCINOMA of the exocrine pancreas is increasing in incidence in the United States and Western Europe (Morgan & Wormsley, 1977). It is difficult to diagnose in a stage at which conventional therapy is likely to be effective, and is almost inevitably fatal. Laboratory studies of this tumour have been limited, by the lack of experimental models, to analysis of surgically excised tissue (Grant *et al.*, 1978), pancreatic and duodenal juice (Allan & White, 1977) or other body fluids from cancer-bearing patients (Chu *et al.*, 1977; Gelder *et al.*, 1978). There is a need for detailed *in vitro* studies, but the difficulty of establishing the tumour in primary culture or as xenografts in immune-deficient animals is reflected by the limited number of successful reports in this field (Lieber *et al.*, 1975; Owens *et al.*, 1976; Yunis *et al.*, 1977; Akagi & Kimoto, 1977; Schmidt *et al.*, 1977; Courtenay & Mills, 1978). This paper reports the establishment and characterization of primary pancreatic exocrine adenocarcinoma in tissue culture, together with xenografts in immune-deficient mice.

### MATERIALS AND METHODS

*Cell culture.*—Tissue from a 1·5 cm, anaplastic and poorly differentiated primary adenocarcinoma in the head of the pancreas in a 56-year-old female was placed in culture medium immediately after removal at operation by pancreaticoduodenectomy. Peripheral tumour was dissected free of surrounding normal tissue, cut into small segments and minced with crossed scalpels in a small volume of fresh medium. Aliquots of this crude cell suspension were evenly dispersed into 25 cm² plastic culture flasks (Falcon) together with a further 4 ml of culture medium. Preliminary studies were carried out using Ham's F12, Eagle's basal essential medium (BEM) or Dulbecco's modification supplemented with 10% foetal calf serum, non-essential amino acids, 1 mM glutamine, 200 u/ml penicillin G, 200 μg/ml streptomycin, 50 μg/ml gentamicin (Flow Laboratories) and 50 μg/ml soya-bean trypsin inhibitor (Sigma Chemical Co.). Flasks were incubated at 37°C in 5%

$CO_2$ in air with medium changes every 3–4 days. Confluent cell monolayers were passaged by treatment with 1·0 ml 0·02% EDTA in Ca- and Mg-free Earle's solution (Flow Laboratories) for 5–10 min at 37°C and reseeded at an initial cell density of $5 \times 10^5$ cells/25 $cm^2$ flask.

*Growth in soft agar.*—1000–10,000 cells suspended in 1 ml supplemented Ham's F12 medium containing 0·3% agar (Gibco Biocult) were layered over a 0·5% agar gel contained in 35 mm Petri dishes and incubated at 37°C in 5% $CO_2$ in air. Colony formation was scored after 1–2 weeks.

*Growth on fibroblast monolayer.*—Early-passaged (4°) diploid human fibroblasts grown to confluency in supplemented Ham's F12 were used as a feeder layer to identify neoplastic cells by their ability to replicate a monolayer of confluent fibroblasts (Lieber *et al.*, 1975). Tumour cells ($5 \times 10^5$) in 5 ml fresh medium were added to the fibroblast monolayer. Plating efficiency was determined by counting colonies after 1–2 weeks.

*Chromosome analysis.*—Fresh medium was added to cultures 1 week after subculture and 15 h before addition of 1 $\mu$g/ml colcemid (Gibco Biocult). Cells were incubated for a further 3 h at 37°C, suspended with EDTA, washed, lysed with 75mM KCl for 10 min and fixed with methanol/acetic acid (3:1). Chromosome preparations were stained with Geimsa, mounted and photographed for counting (Schwarzacher & Wolf, 1974). 100 cells were analysed at 2 different passages.

*Autoradiography.*—Cells cultured in fresh medium were exposed to 0·5 $\mu$Ci/ml $^3$H-thymidine (28 Ci/mmol, Radiochemical Centre, Amersham) for 12 h at 37°C, washed in phosphate-buffered saline (PBS) fixed with methanol/acetic acid and 5% TCA, and processed for autoradiography using Ilford K2 liquid emulsion (Ilford Ltd., Cheshire). Autoradiographs were developed after 7–14 days and stained with Leishman's. The labelling index was calculated from the number of radioactively labelled cells in 200 cells.

*Xenografts.*—Female outbred congenitally athymic nu/nu mice about 2 months old were used in these studies. Mice were bred and maintained in sterile conditions in a negative pressure isolater and remained healthy and disease-free throughout.

Tissue was obtained immediately after operative removal of a moderately well-differentiated adenocarcinoma in the body of the pancreas of a 60-year-old female (IR) and a poorly differentiated adenocarcinoma in the head of the pancreas in a 63-year-old female (WB). The tumour tissue was washed in culture medium, minced with scissors and homogenized by passage through a syringe with 16-gauge needle. Tumour mince (0·05 ml) was transplanted s.c. in each of 2 thoracic and inguinal mammary sites per mouse. Pancreatic tumour cells growing *in vitro* were harvested by EDTA treatment and $4 \times 10^6$ (6th passage) or $1 \times 10^7$ (8th passage) cells were injected s.c. in a similar manner. When tumours at one or more sites measured ~1·5 cm in 2 dimensions the mice were killed, tumours excised, minced, homogenized and transplanted. Histological sections of transplanted tumours were fixed in neutral buffered formalin and stained with haemotoxin and eosin.

*Assay of pancreas-specific and intracellular enzymes.*—An electrophoretic study of exportable pancreatic enzymes (chymotrypsin, trypsin, carboxypeptidase A, elastase and their related zymogens, lipase, and DNase I) together with DNase II, carbonic anhydrase, alkaline phosphatase and a number of other intracellular carbohydrate-metabolizing enzymes, was performed on the cell line and xenografts according to methods previously described for normal pancreas and native primary tumour tissue (Grant *et al.*, 1978). Cells were washed with PBS, lysed with an equal volume of distilled water and assayed directly. Xenografts were homogenized in an equal volume of water or 70 mM sodium succinate (pH 8·6) and the homogenate centrifuged at 5000 *g* to obtain a supernatant for assay.

### RESULTS

### In vitro *cell culture*

One of 9 pancreatic tumour specimens was successfully cultured *in vitro*. Within a few days of dispersal of fragments of this tumour into culture flasks, small areas of epithelial-cell outgrowth were observed surrounding adherent tissue. Cell growth was found to be most successful in supplemented Ham's F12. Dulbecco's and BEM both encouraged more fibroblast growth. After 2 months large epithelial islands could be seen; some persistent fibroblasts were removed by trypsinization. There-

FIG. 1.—Monolayer of pancreatic exocrine adenocarcinoma cells growing in supplemented Ham's F12 (×150)

FIG. 2.—Pancreatic tumour cells growing as a single epithelial cell colony on a monolayer of early diploid human fibroblasts (×150)

FIG. 3.—Histological sections of; (a), Primary poorly-differentiated pancreatic adenocarcinoma from which the cell line was established and (b), xenografts derived from injection of the pancreatic tumour-cell line into a nude mouse (× 75).

after, the epithelial cells grew to confluency and were passaged by EDTA treatment alone. The cells grew as an epitheloid monolayer without fibroblast contamination, and have undergone 20 serial transfers to date (Fig. 1). Plating efficiency increased with passage number and initial cell density; at least 30% of the cells now re-adhere after EDTA treatment. Doubling time decreased with increasing passage, being 48 h at 6th passage and 36 h at 11th passage, but now appears to have stabilized. A confluent monolayer is achieved after 7–10 days in culture. At confluency, density-dependent inhibition of cell growth is not found. As shown by their uptake of $^3$H-thymidine (labelling index 48–53%) 50% of the adherent cells are still actively growing, and this may be an underestimate since the cell cycle is probably greater than 12 h. Maintenance of confluent cultures results in substantial loss of adherence, with increasing numbers and proportion of cells in the supernatant.

Throughout subconfluency the cell line also exhibits a constant proportion of cells free in suspension (floaters) which account for 10–15% of the total cell population. Over 50% of these floaters are viable, as judged by trypan-blue exclusion, but mitosis is rarely seen, and the uptake of [$^3$H]-TdR is only one third of that found with cells in monolayer. Less than 1% of these "floaters" will form colonies if reseeded into new flasks, but the cells which do grow appear to have the same characteristics as the original monolayer.

Evidence of tumour origin was provided by a modal chromosome number of 62 at 8th and 12th passage, 80% of the cells having a chromosome number 60–65, 10% being tetraploid or polypoid. Their tumourigenicity was indicated by growth on a confluent monolayer of early-passage fibroblasts with a plating efficiency (PE) of at least 30% (Fig. 2), and growth in soft agar with a PE of 2%. Normal diploid human fibroblasts grew in both of these conditions with PEs of 0·001% and 0·005% respectively. The adherent monolayer cells also grew as solid tumours in nude mice after an initial lag phase of 5 weeks; no growth has so far been obtained with floating cells or fibroblasts. There are similarities in the histological appearances of the mouse xenografts and the original primary pancreatic tumour (Fig. 3).

*Xenografts from primary human tissue*

Two out of 3 tissue samples of primary pancreatic exocrine adenocarcinoma have been established directly as xenografts (Fig. 4); both have been transplanted with a greater than 90% "take" rate in successive passages. Although the 2 lines have different growth rates they show similar characteristics: a prolonged latent period after the initial implantation

FIG. 4.—Congenitally athymic nude mouse bearing xenografts derived directly from primary pancreatic adenocarcinoma.

TABLE I.—*Growth characteristics of xenografts established directly from primary pancreatic adenocarcinoma*

| Passage No. | 1 | 2 | 3 | 4 | 5 |
|---|---|---|---|---|---|
| Patient IR ♀ aged 63 | | | | | |
| Lag phase (weeks) | 17 | 3 | 2 | | |
| Weeks to transplant | 26 | 17 | 9 | | |
| Patient WB ♀ aged 60 | | | | | |
| Lag phase (weeks) | 8 | 4 | 2 | 2 | 2 |
| Weeks to transplant | 13 | 10 | 8 | 9 | 7 |

TABLE II.—*Electrophoretic characterization of pancreas-specific enzymes in normal pancreas, pancreatic carcinoma cancer cell line, xenografts and mouse mammary gland*

| | | | Tissues | | | | |
|---|---|---|---|---|---|---|---|
| Enzyme | Substrate* | Electro-phoretic mobility | Normal pancreas | Pancreatic carcinoma | Pancreatic cancer cell line | Pancreatic cancer xenografts | Mouse mammary gland |
| Trypsin | BANA | ($\alpha_1$) $\beta$ | ++++ | (+) | — | — | — |
| Trypsinogen | BANA | $\alpha_1$ $\beta$ | ++++ | (+) | — | — | — |
| Chymotrypsin/ogen† | BSPP | $\beta$ ($\gamma$) | ++++ | (+) | — | — | — |
| Chymotrypsin/ogen‡ | BSPP | $\alpha_1$ | (+) | — | — | +++ | +++ |
| Carboxypeptidase A | CNP | $\beta_1$ | ++++ | (+) | — | — | — |
| Procarboxypeptidase A | CNP | $\alpha_1$ | ++++ | (+) | — | — | — |
| Lipase | $\alpha$-Napthy-laurate | $\beta_2$ | +++ | (+) | — | — | — |
| Elastase | Elastin | $\gamma$ | +++ | — | — | — | — |
| DNase I (pancreas-specific) | DNA | $\alpha_2$ $\beta$ | +++ | (+) | — | + | — |
| DNase II (lysosomal) | DNA | $\beta$ | +++ | +++ | ++ | +++ | +++ |

* BANA—N-benzoyl-DL-arginine.
BSPP—benzosalicylanilide-$\beta$-phenyl proprionate.
CNP—N-carbo-$\beta$-napthoxy-DL-phenylalanine.
† —activity in normal pancreas.
‡ —enzyme in mouse tissue.
+ → +++ degree of enzyme activity.
Brackets indicate weak activity.

followed by slow growth. On serial transplantation the latent period shortens and the growth rate increases (Table I). The histological appearances of the xenografts were maintained throughout transplantation (Fig. 5).

*Enzyme profile of cell line and xenografts*

An electrophoretic study of a number of pancreatic enzymes normally exported by acinar cells established their absence from the tumour cell line (Table II) in agreement with the low enzyme levels previously found in native primary pancreatic cancer tissue (Grant *et al.*, 1978). Chymotrypsin-like activity which cleaved the synthetic substrate benzosalicylanilide-$\beta$-phenyl proprionate was detected in the extracts of the xenografts, but had a different electrophoretic mobility to human pancreatic chymotrypsin, and was found to come from surrounding mouse mammary tissue. Human pancreatic DNase I activity was only detected in xenograft extracts; non-specific lysozomal DNase II could be detected in all tissues tested.

Intracellular enzymes glucose 6 phosphate dehydrogenase (G6PD), 6-phosphogluconate dehydrogenase (6PGD), phosphoglucose isomerase (PGI), and phosphoglucomutase (PGM) were retained in the cell line and xenografts, together with carbonic anhydrase and alkaline phosphatase, as the most common human phenotypes (Table III).

TABLE III.—*Intracellular enzyme phenotypes in normal pancreas, tumour tissue, cancer cell line and xenografts*

| | Tissues | | | |
|---|---|---|---|---|
| Enzyme | Normal pancreas | Pancreatic carcinoma | Pancreatic cancer cell line | Xenograft |
| G6PD | B | B | B | B |
| 6GPD | A | A | A | A |
| $PGM_1$ | 1 | 1 | 1 | 1 |
| $PGM_2$ | 1 | 1 | — | 1 |
| PGI | 1 | 1 | 1 | 1 |
| Alk. Phosphatase | $\alpha_2$ | $\alpha_2$ ($\alpha_1$) | $\alpha_2$ | $\alpha_2$ |
| Carbonic Anhydrase | I, II | I, II | I, II | I, II |

### DISCUSSION

The difficulty of establishing pancreatic exocrine adenocarcinoma in tissue culture (one out of 9 attempts) is in general agree-

(a)

(b)

FIG. 5.—Histological sections of pancreatic tumour tissue established directly as xenografts. (a), primary tissue; (b), at 1st transplantation; (c), at 4th transplantation ($\times$ 100).

FIG. 5(c)

ment with the experience of others (Lieber *et al.*, 1975). The pancreatic tumour established *in vitro* was small (1·5 cm), anaplastic, and contained a higher proportion of tumour cells to infiltrating normal host cells than in the other more advanced tumours removed; thus the tumour-cell density at the initial plating was likely to have been greatest. The lack of differentiation in this tumour was indicated by the abundance of mitotic figures and the conspicuous nuclear pleomorphism. This is in agreement with the concept that the ability of cells to grow in tissue culture is inversely related to their stage of differentiation. The degree of differentiation of the tumour does not, however, appear to affect the formation of xenografts to the same extent. The success rate in the present study was comparable to that recently reported by Schmidt *et al.* (1977).

Previous studies in this laboratory (Grant *et al.*, 1978) have shown that extracts of native pancreatic tumour tissue do not contain pancreatic secretory enzyme activity. The absence of these enzymes from the cultured cells or xenografts further supports the lack of acinar-cell differentiation. The cultured cells, however, did contain appreciable carbonic anhydrase of the normal human phenotype, but in the absence of a suitable control cell population it is not possible to infer a commitment to differentiate into ductular epithelium, as might be normally expected to occur during embryonic development.

An unusual characteristic of the tumour-cell line we have established is the presence of a proportion of viable floating cells associated with the monolayer, a feature which has previously been described for mouse mammary tumours *in vitro* (Hosick, 1976). It suggests an exaggerated loss of specific cell adhesion, which may be associated with the process of metastasis clinically. The patient unfortunately developed widespread diffusely infiltrating osseous and visceral tumour within 6 months of resection, despite the absence of microscopic lymphnode involvement or other evidence of extra-pancreatic progression at surgery. Neither the xenografts from this cell line nor those from direct primary tumour implantation, however, showed the ability to metastasize in nude mice.

The histological similarities between each xenograft and its tumour of origin, together with the continued presence of intracellular enzymes of the expected human phenotype, suggests that the cultured tumour cells have preserved many of their original characteristics. These *in vitro* and *in vivo* models should provide a representative source of tumour components uncontaminated by the normal tissue inevitably present in native tumours.

We are most grateful to Professor K. Hellmann for his valuable discussion and support. This work was funded in part by a grant from the Cancer Research Campaign to the Department of Surgery, St George's Hospital Medical School.

### REFERENCES

ALLAN, B. J. & WHITE, T. T. (1977) Bile, pancreatic cancer and the activation of pancreatic juice. *Biochem. Biophys. Res. Commun.*, **79**, 485.

AKAGI, T. & KIMOTO, T. (1977) Establishment and characteristics of a human pancreatic cancer cell-line (HGC-25). *Acta Pathol. Jpn.*, **27**, 51.

CHU, T. M., HOLYOKE, E. D. & DOUGLASS, H. O. (1977) Isolation of a glycoprotein antigen from ascites fluid of pancreatic carcinoma. *Cancer Res.*, **37**, 1525.

COURTENAY, V. D. & MILLS, J. (1978) An *in vitro* colony assay for human tumours grown in immune-suppressed mice and treated *in vivo* with cytotoxic agents. *Br. J. Cancer*, **37**, 261.

GELDER, F. B., REESE, C. J., MOOSA, A. R., HALL, T. & HUNTER, R. (1978) Purification, partial characterisation and clinical evaluation of a pancreatic oncofetal antigen. *Cancer Res.*, **38**, 313.

GRANT, A. G., MCGLASHAN, D. & HERMON-TAYLOR, J. (1978) A study of pancreatic secretory and intracellular enzymes in pancreatic cancer tissue, other gastrointestinal cancers, normal pancreas and serum. *Clin. Chim. Acta*, **90**, 75.

HOSICK, H. (1976) Spontaneous cell loss during growth of postconfluent primary cultures from mammary adenocarcinomas. *Cancer. Res.*, **36**, 3126.

LIEBER, M., MAZZETTA, J., NELSON-REES, W., KAPLAN, M., TODARO, G. (1975) Establishment of a continuous tumour cell-line (PANC-1) from human carcinoma of the exocrine pancreas. *Int. J. Cancer*, **15**, 741.

MORGAN, R. G. H. & WORMSLEY, K. G. (1977) Cancer of the pancreas. *Gut*, **18**, 580.

OWENS, R. B., SMITH, H. S., NELSON-REES, W. A. & SPRINGER, E. L. (1976) Epithelial cell cultures from normal and cancerous human tissue. *J. Natl Can. Inst.*, **56**, 843.

SCHMIDT, M., DESCHNER, E. E., TALER, H. T., CLEMENTS, L. & GOOD, R. A. (1977) Gastrointestinal cancer studies in the human to nude mouse heterotransplant system. *Gastroenterology*, **72**, 829.

SCHWARZACHER, H. G. & WOLF, U. (Eds) (1974) *Methods in Human Cytogenetics*, Berlin: Springer-Verlag.

YUNIS, A. A., ARIMURA, G. K. & RUSSIN, D. J. (1977) Human pancreatic carcinoma (MIA PaCa-2) in continuous culture: sensitivity to asparaginase. *Int. J. Cancer*, **19**, 128.